# Establishment of a Novel Short Tandem Repeat Typing Method for *Exophiala dermatitidis*

**DOI:** 10.1007/s11046-023-00825-y

**Published:** 2024-01-17

**Authors:** Hamide Zoqi, Dirk Schmidt, Ludwig Sedlacek, Peter-Michael Rath, Joerg Steinmann, Lisa Kirchhoff

**Affiliations:** 1 Institute of Medical Microbiology, ECMM Center of Excellence in Clinical and Laboratory Mycology and Clinical Studies (Diamond Status), University Hospital Essen, University of Duisburg-Essen, 45122 Essen, Germany; 2 Institute of Medical Microbiology and Hospital Epidemiology, Medical School Hannover (MHH), Hannover, Germany; 3 Institute of Clinical Microbiology, Infectious Diseases and Infection Control, Klinikum Nürnberg, Paracelsus Medical University, 90419 Nuremberg, Germany

**Keywords:** *Exophiala dermatitidis*, Microsatellite PCR, Genotyping, Short tandem repeats, Cystic fibrosis

## Abstract

The opportunistic black yeast-like fungus *Exophiala dermatitidis* frequently colonizes the respiratory tract of cystic fibroses (CF) patients. Additionally, it can cause superficial, systemic, and cerebral forms of phaeohyphomycoses. The objective of this study was to develop and apply a microsatellite or short tandem repeat (STR) genotyping scheme for *E. dermatitidis*. In total, 82 *E. dermatitidis* isolates from various geographic origins (environmental = 9, CF = 63, invasive isolates = 9, melanin-deficient mutant = 1) were included in this study. After next-generation sequencing of a reference strain and sequence filtering for microsatellites, six STR markers were selected and amplified in two multiplex PCR reactions. The included isolates were discriminated in a genetic cluster analysis using the Pearson algorithm to reveal the relatedness of the isolates. The *E. dermatitidis* isolates clustered on basis of both, their source and their origin. The invasive isolates from Asia were unrelated to isolates from CF. Nearly all environmental isolates were grouped separately from patients’ isolates. The Simpson index was 0.94. In conclusion, we were able to establish a STR genotyping scheme for investigating population genomics of *E. dermatitidis.*

## Introduction

The incidence of infections with fungi increased in the last years posing new challenges to health care professionals. Black yeast-like fungi are opportunistic and rarely found as pathogens in clinical specimen. The most prominent member of the group of black yeast-like fungi is *Exophiala dermatitidis. Exophiala* belongs to the *Ascomycotina* order *Chaetothyriales*. The taxonomic classification of *E. dermatitidis* has undergone revisions over time, and it is currently classified under the genera *Exophiala,* formerly known as *Wangiella* [1].

*E. dermatitidis* exhibits distinct characteristics, among others the melanized and thick cell walls, being responsible for high resistances to environmental stress, including temperature and salt concentrations, and the capability to switch between conidial and hyphal forms. The presence of melanin in the cell wall contributes to the fungus's virulence and enhances its resistance to host defense mechanisms and antifungal treatments [2, 3].

In nature, *E. dermatitidis* is ubiquitously distributed in various environments, including extreme natural habitats, hydrocarbon-rich artificial settings like steam baths and bathrooms, decaying organic matter, and even dishwashers [4]. Little is known about the natural habitat and the transmission routes of *E. dermatitidis* [2]. It is suggested that the natural habitat of *E. dermatitidis* is the warm and wet tropics, as *E. dermatitidis* is regularly found in environments with high temperatures, high humidity and pH changes. One potential transmission route for *E. dermatitidis* is through aerosol inhalation from household dishwashers, although household-acquired colonisations seems to be rare and predominantly observed in individuals with conditions such as cystic fibrosis (CF) or immunosuppression [4].

In the Western countries, *E. dermatitidis* is mostly isolated from the sputa of CF patients, only once in a while with clinical relevance. Furthermore, *E. dermatitidis* rarely acts as the primary causative agent of fungal infections affecting immunocompromised individuals [2]. Additionally, *E. dermatitidis* has been described to cause invasive infections of the central nervous system of otherwise healthy individuals with Asian origin. In systemic and invasive cases, it has been associated with mortality rates ranging from 25 to 80% [5]. However, *E. dermatitidis* is also described to cause cutaneous and superficial infections in both humans and animals [6].

Advancements in various methods, including molecular methods, have shown promising results in the identification of *E. dermatitidis*. Techniques such as matrix-assisted laser desorption-ionization time of flight mass spectrometry (MALDI-TOF MS), PCR, and ITS sequencing have been utilized for rapid and accurate identification [7–9]. The internal transcribed spacer region (ITS) has been particularly useful as a barcode marker for distinguishing black yeasts, including *E. dermatitidis*, and can even differentiate between different subtypes [10].

However, limitations include incomplete reference databases for black yeast species and challenges in distinguishing closely related species [7, 11]. This led us to conduct a thorough assessment of current typing techniques and select novel microsatellite markers to differentiate *E. dermatitidis* and to perform cluster analysis of various *E. dermatitidis* isolates. This is the first study on genotyping using short tandem repeat markers for discrimination of *E. dermatitidis* isolates from various sources.

## Materials and Methods

### Study Outline

After identifying short tandem repeat markers and establishing a microsatellite PCR, we applied the new method to a total of 82 clinical isolates of *E. dermatitidis*. A genotypic cluster analysis was performed.

### Isolates

The study did not include patient’s details and did not result in additional constraints for the patients. All data (fungal strains) were anonymously analyzed without patients' consent due to the retrospective nature of the study. All procedures and methods were carried out in accordance with approved guidelines.

Isolates were predominantly collected from patient’s specimen, mainly CF sputa, as well as from environmental sources across different countries. All isolates are listed with their origin in Table 1. In case of the isolates obtained from CF sputa, for three patients, serially isolated *E. dermatitidis* were collected over time and included. These isolates are marked by patients' ID (a, b, c) in the table below.

**Table 1:** List of included *Exophiala dermatitidis* isolates

Isolate	Reference ID	Description	Source	City/country of origin	Serial isolate
F111	CBS120574	Environmental	Sauna room	Thailand	–
F116	CBS 123467	Clinical	Human, CF	China	–
F101	CBS 549.90	Clinical	Human, CF	Germany	–
F102	CBS 748.88	Clinical	Human, CF	Norway	–
F103	CBS 148.90	Clinical	Human, CF	Germany	–
F106	CBS 153.90	Clinical	Human, CF	Germany	–
F104	CBS 213.90	Clinical	Human, CF	Germany	–
F105	CBS 156.90	Clinical	Human, CF	Germany	–
F118	CBS 116372	Clinical	Human, invasive	Japan	–
F120	CBS 579.76	Clinical	Human, invasive	Japan	–
F117	CBS 109154	Clinical	Human, invasive	South Korea	–
F114	CBS120435	Environmental	Steam bath	Thailand	–
F112	CBS120479	Environmental	Air	Germany	–
F115	CBS 578.76	Clinical	Human, invasive(chromomycosis)	Taiwan	–
F119	CBS 577.76	Clinical	Human, invasive	Taiwan	–
F 05	CBS 149.90	Clinical	Human, CF	Aachen, Germany	–
F 39	CBS 154.90	Clinical	Human, CF	Aachen, Germany	–
F 40	CBS 207.35	Clinical	Human, invasive	Osaka, Japan	–
F 41	CBS 552.90	Clinical	Human, CF	Aachen, Germany	–
F 82	CBS 120550	Environmental	Steam bath	Austria	–
F 83	CBS 120546	Clinical	Human	Greece	–
F 84	CBS 120429	Clinical	Human, CF	Finland	–
F 85	CBS 109143	Environmental	Shower	Laren, The Netherlands	–
F 86	CBS 109148	Clinical	Human	Human faeces, The Netherlands	–
F 88	CBS 109142	Environmental	Berry	The Netherlands	–
F 91	CBS 739.87	Environmental	Lager beer	Ireland	–
F 92	CBS 109153	Clinical	Human	Oulu, Finland	–
1872		Clinical	Human, CF	Essen, Germany	c
1873		Clinical	Human, CF	Essen, Germany	a
1874		Clinical	Human, CF	Essen, Germany	a
1883		Clinical	Human, CF	Essen, Germany	–
1908		Clinical	Human, CF	Hannover, Germany	–
1909		Clinical	Human, CF	Hannover, Germany	–
1910		Clinical	Human, CF	Hannover, Germany	–
1911		Clinical	Human, CF	Hannover, Germany	–
1912		Clinical	Human, CF	Hannover, Germany	–
1913		Clinical	Human, CF	Hannover, Germany	–
1915		Clinical	Human, CF	Hannover, Germany	–
1916		Clinical	Human, CF	Hannover, Germany	–
1917		Clinical	Human, CF	Hannover, Germany	–
1919		Clinical	Human, CF	Hannover, Germany	–
1920		Clinical	Human, CF	Hannover, Germany	–
1921		Clinical	Human, CF	Hannover, Germany	–
1922		Clinical	Human, CF	Hannover, Germany	–
1923		Clinical	Human, CF	Hannover, Germany	–
1924		Clinical	Human, CF	Hannover, Germany	–
1925		Clinical	Human, CF	Hannover, Germany	–
1926		Clinical	Human, CF	Hannover, Germany	–
1927		Clinical	Human, CF	Hannover, Germany	–
1928		Clinical	Human, CF	Hannover, Germany	–
1929		Clinical	Human, CF	Hannover, Germany	–
1930		Clinical	Human, CF	Hannover, Germany	–
1931		Clinical	Human, CF	Hannover, Germany	–
1932		Clinical	Human, CF	Hannover, Germany	–
1933		Clinical	Human, CF	Hannover, Germany	–
1935		Clinical	Human, CF	Hannover, Germany	–
1936		Clinical	Human, CF	Hannover, Germany	–
1937		Clinical	Human, CF	Hannover, Germany	–
1938		Clinical	Human, CF	Hannover, Germany	–
1939		Clinical	Human, CF	Hannover, Germany	–
1940		Clinical	Human, CF	Hannover, Germany	–
1942		Clinical	Human, CF	Hannover, Germany	–
1943		Clinical	Human, CF	Hannover, Germany	–
1947		Environmental	Dish washer	Essen, Germany	–
1948		Environmental	Dish washer	Essen, Germany	–
1952		Clinical	Human, CF	Essen, Germany	c
2011		Clinical	Human, CF	Essen, Germany	c
2021		Clinical	Human, CF	Essen, Germany	c
2094			Melanin deficient Mutant Mel^−3^, derived from ATCC 34100,	Aachen, Germany	–
2128		Clinical	Human, CF	Essen, Germany	–
2565		Clinical	Human, CF	Essen, Germany	–
2566		Clinical	Human, CF	Essen, Germany	b
2567		Clinical	Human, CF	Essen, Germany	b
2569		Clinical	Human, CF	Essen, Germany	b
2570		Clinical	Human, CF	Essen, Germany	b
2571		Clinical	Human, CF	Essen, Germany	b
2572		Clinical	Human, CF	Essen, Germany	b
2574		Clinical	Human, CF	Essen, Germany	b
2575		Clinical	Human, CF	Essen, Germany	b
2578		Clinical	Human, CF	Essen, Germany	b
2579		Clinical	Human, CF	Essen, Germany	b
2580		Clinical	Human, CF	Essen, Germany	–

All isolates were cultured on malt extract agar (Life Technologies GmbH, Darmstadt, Germany) and incubated at a temperature of 35°C for a duration of 48 h. Identification of the isolates relied on a combination of macroscopic and microscopic morphology evaluation. In case of uncertainty additionnally sequencing of the internal transcribed spacer region 1 (ITS1) was performed [12].

### DNA Extraction

DNA was extracted using the Maxwell16 nucleic acid extraction instrument (Promega, Mannheim, Germany) and the Maxwell16 Tissue LEV Total RNA Purification Kit. Several colonies were inoculated in sterile water and vigorously shaken in a 2-mL innuSPEED Lysis Tube B (Analytic Jena, Jena, Germany) three times at 2000× *g* for 50 s each using a MagNA Lyser (Roche Diagnostics, Basel, Switzerland). After centrifugation at 8600× *g* for 30 s, the supernatant was transferred to the extraction cartridge. DNA was eluted in 50 μL of nuclease-free water. DNA concentrations were determined using the NanoDrop 1000 instrument (PeqLab Biotechnologie GmbH, Erlangen, Germany).

### Identification of Microsatellite Primers and Microsatellite PCR

Next-generation sequencing and sequence filtering for microsatellites were conducted by ecogenics GmbH (Balgach, Switzerland) using the *E. dermatitidis* reference strain CBS 550.90. In total, 286 potential primer pairs were identified (data not shown).

The Illumina TruSeq nano library was analyzed on an Illumina MiSeq sequencing platform using a nano v2 500 cycles sequencing chip (Illumina, CAL, USA). The resulting paired-end reads which passed Illumina’s chastity filter were subject to de-multiplexing and trimming of Illumina adaptor residuals. Subsequently the quality of the surviving reads was checked with the software FastQC v0.117. In a next step the paired end reads were merged with the software USEARCH v10.0.240 to in-silico reform the sequenced molecule. The resulting merged reads were screened with the software Tandem Repeats Finder, v4.09. After this process, 7′409 merged reads contained a microsatellite insert with a tetra- or a trinucleotide of at least 6 repeat units or a dinucleotide of at least 10 repeat units. Primer design was performed with primer 3. Suitable primer design was possible in 5′848 microsatellite candidates. Primers were chosen according to the size of the amplification product in order to be able to perform multiplex pcr and according to the motif (motif variation).

Multiplex PCR was carried out using the Taq PCR Core Kit (Qiagen, Hilden, Germany) with three different primer sets. Each primer set consisted of three primer pairs, with one primer labeled with FAM, VIC/HEX, and NED markers, respectively (Table 2). The reaction mixture consisted of 10 μL of reaction buffer, 2 μL of dNTP mix, 2 μL of each primer per set, 0.8 μL of Taq polymerase, and 55.2 μL of nuclease-free water, resulting in a total volume of 80 μL. Subsequently, 20 μL of the reaction mix was combined with 5 μL of DNA at a concentration of 1 ng/μL. The microsatellite analysis took place on an abi3130 sequencing instrument (Life Technologies, Germany) in a total volume of 10 μL.

**Table 2:** Sequences of six primer pairs used for microsatellite PCR

STR	Forward primer sequence	Reverse primer sequence	MasterMix
1A (CCA)	5′-ACGAGGATAGGGTTGCCTTG-3′	5′-TAAGGGCGTGTTCACTGGAG-3′	1
2B (CT)	5′-CAGGTTGAACATTCACGGGG-3′	5′-TGTCAAACTGCTCGATTGCG-3′	2
2C (TGG)	5′-TACTAGCAGGGCTCGATGTC-3′	5′-CTGGCAGATCGTCTTTTCCG-3′	2
3C (GAA)	5′-ACATCAATGCAAGCCTCGAC-3′	5′-TGTAGCTGACAACGTCCTCC-3′	2
4A (GAC)	5′-AACTTCTTGAAACAGGGCGG-3′	5′-TCGCTAGGGGTTGGGATTTC-3′	1
5B (AG)	5′-TGGATACGACAAGGGCTGTG-3′	5′-ACTAGTATGGGCCGGCAAG-3′	1

*STR* short tandem repeat

For analysis, 2 μL of each amplification product was mixed with 0.5 μL of GeneScan 1200 LIZ size standard and 7.5 μL of Hi-Di formamide (both supplied by Life Technologies). After denaturation at 92 °C for 2 min, the reaction was rapidly cooled on ice. Based on the fragment size, the DNA samples were subsequently classified into distinct genotypes.

### Statistical Analysis

The analysis of microsatellite data was conducted using the R program version 4.2.2 (2022-10-31). The cluster dendrograms are created using the R program with the Pearson algorithm to visualize the similarities and differences among the samples, allowing for the identification of distinct clusters based on their geographic origin and sources (CF, invasive, environmental sources).

A robust approach was adopted to quantify the epidemiological cutoff values (ECVs/ECOFFs) for each average primer value, utilizing the 95th percentile method. This statistical approach, performed in R, allowed the calculation of upper thresholds for each primer value. This statistical procedure facilitated the derivation of ECVs, acting as upper thresholds for attribute values. Then, Isolates were classified based on primer values relative to the calculated ECVs. A classification system was implemented to categorize isolates as “related” or “unrelated,” which was integrated into the dataset. Isolates with primer values below the ECV were categorized as “related,” whereas those surpassing the ECV were classified as “unrelated.”

The discriminatory power of the STR typing method was mathematically defined by calculating the Simpson index of diversity (D): $D=1-\frac{1}{N\left(N-1\right)}\sum_{j=1}^{s}{n}_{j}\left({n}_{j}-1\right)$, where N is the total number of isolates, s is the total number of clades, and n_j_ is the number of isolates belonging to the jth type. A D value of 1 indicates good discriminatory power of the method whereas a D value of 0.0 indicates that all included isolates are defined as identical by this method.

## Results

**Fig. 1:**
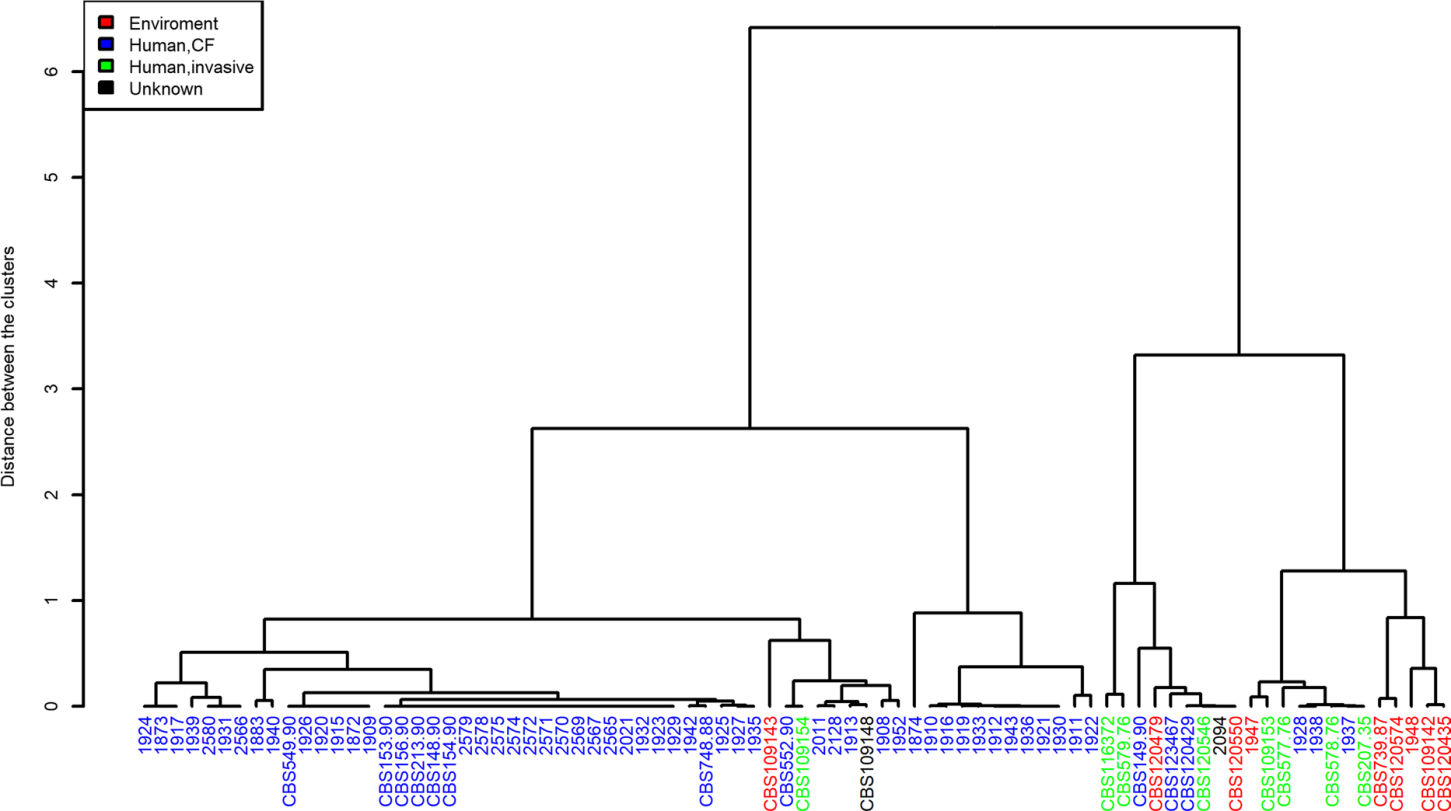
Dendrogram of *E. dermatitidis* isolates from various sources with marked origin. Red: Environment; Blue: Human, CF; Green: Human, invasive; black: unknown

A cluster analysis with Pearson algorithm was performed. The analysis revealed a distinct cluster for the invasive isolates from patients with Asian origin, marked in Fig. 1 in green. In addition, the environmental isolates, marked in red, cluster with one exception: Isolate CBS109143, obtained from a shower in the Netherlands, clustered more likely with human isolates from CF than with the other isolates from environmental origin (Fig. 1).

**Fig. 2:**
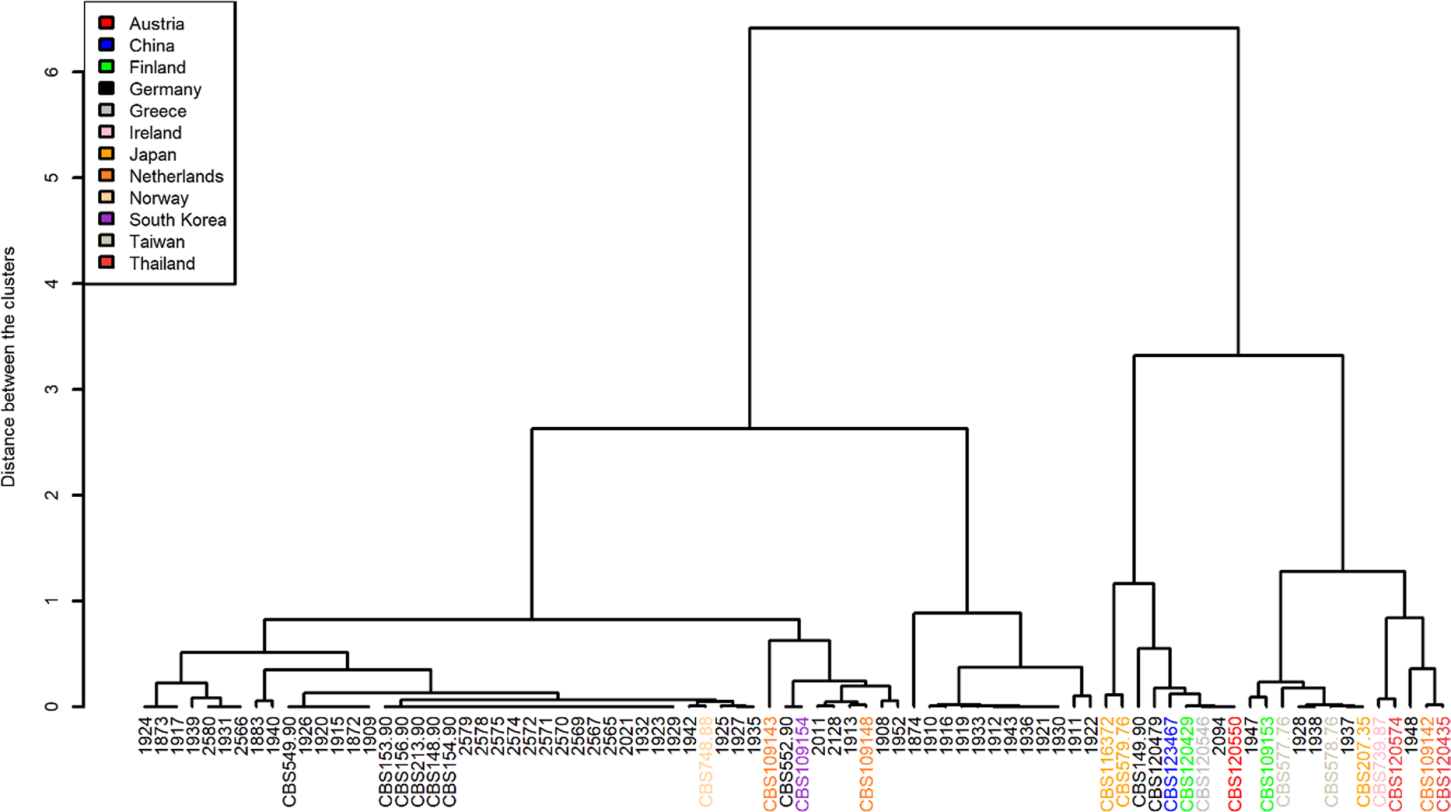
Dendrogram of *E. dermatitidis* isolates from various sources with marked country of origin

Serial isolates from patients a and c were scattered across the dendrogram while the serial isolates from patient b were mainly of the same genotype. The dendrogram built for the cluster analysis of *E. dermatitidis,* in recognition of their origin, showed closely related isolates from the same or similar origin (Fig. 2). More than one cluster consists solely of isolates from Germany. In addition, the Greek isolates cluster together.

Based on the calculated ECVs, a cut-off value of 95% was used to distinguish between related and unrelated strains. As a result, 77 isolates exhibited genetic relatedness and were classified as related strains. On the other hand, five isolates (F111, F88, F114, F05, F120) showed lower genetic relatedness and were classified as unrelated strains. In addition, strains were named related even though they were from distinct sources and geographical origins.

The calculated Simpson diversity index was 0.94, indicating that the typing method of microsatellite PCR for *E. dermatitidis* is suitable for the discrimination of unrelated isolates.

## Discussion

We here performed for the first time a genetic cluster analysis via STR of 82 *E. dermatitidis* isolates from various origins. The included strain collection is diverse and the isolates were from three sources: 9 environmental isolates and 73 clinical isolates, among them 63 CF and 9 invasive isolates as well as one melanin-deficient mutant (mel^−3^ mutant). The data showed distinct cluster for isolates from different origins and sources. The CF isolates cluster together, although no person-to-person transfection is described yet and the suggested source of colonization is the environment. The invasive isolates from Asian and otherwise healthy patients formed distinct clusters. Additionally, the country of origin was influencing the genetic cluster. The distinct strains differ each in more than one STR from another, some even in all.

Fingerprinting of *E. dermatitidis* isolates has been performed with other methods before. However, these analyses were done solely for a set of strains from CF patients sputa [13, 14]. Rath et al. included eleven strains of *E. dermatitidis* from which ten were isolated from CF patients and one was a reference strain from an invasive infection from Japan. In this study from 1997, different methods were applied, none of them being microsatellite PCR. The authors recommend rather the application of fatty acid methyl ester (FAME) profiles and random amplification of polymorphic DNA (RAPD) analysis than assimilation tests. However, each of the results showed a separation of the included Japanese strain from the European CF isolates [13], which is comparable to the here obtained results.

Additionally, Packeu et al*.* performed molecular typing using RAPD of *E-dermatitidis* isolates from patients with CF. They included 71 isolates from 13 patients. They did not find genetic clustering of isolates according to their geographical origin, the date of isolation or their antifungal susceptibility [14]. Packeu et al*.* further included as well serially isolated strains in RAPD analysis. They detected for the majority of the sequential isolates a distribution in patient specific clusters only with a few exceptions [14]. In contrast to these findings, via the microsatellite approach we did see a clustering of isolates from geographical closeness. In this study, basing on the genotypes of the serial isolates, it can be hypothesized that patients a and c got recolonized and the isolates were replaced. All three patients were CF patients.

The here developed molecular typing method showed good discriminatory power with a Simpson index of diversity with 0.94, demonstrating the STR typing being capable to discriminate between most of the *E. dermatitidis* isolates. The method could find application in the genetic analysis of *E. dermatitidis* infection outbreaks, e.g. on clinical wards, as described to took place in the US in 2002 [15] and 2016 [16]. However, there are limitations of this method as relatedness has been detected here for isolates from different geographical origins as well as from differing sources e.g. patients, CF sputa and environment.

## Conclusion

We here developed a novel short tandem repeat scheme for molecular typing of *E. dermatitidis* isolates from various origin, demonstrating geographical and source specific genetic clustering.

## References

[1] Hohl PE, Holley HP, Prevost E, Ajello L, Padhye AA (1983). Infections due to *Wangiella dermatitidis* in humans: report of the first documented case from the United States and a review of the literature. Rev Infect Dis.

[2] Kirchhoff L, Olsowski M, Rath PM, Steinmann J (2019). *Exophiala dermatitidis*: key issues of an opportunistic fungal pathogen. Virulence.

[3] Kirchhoff L, Olsowski M, Zilmans K, Dittmer S, Haase G, Sedlacek L (2017). Biofilm formation of the black yeast-like fungus *Exophiala dermatitidis* and its susceptibility to antiinfective agents. Sci Rep.

[4] Zupančič J, Novak Babič M, Zalar P, Gunde-Cimerman N (2016). The black yeast *Exophiala dermatitidis* and other selected opportunistic human fungal pathogens spread from dishwashers to kitchens. PLoS ONE.

[5] Song Y, Laureijssen-van de Sande WWJ, Moreno LF, Gerrits van den Ende B, Li R, de Hoog S (2017). Comparative ecology of capsular *Exophiala* species causing disseminated infection in humans. Front Microbiol.

[6] Zeng JS, Sutton DA, Fothergill AW, Rinaldi MG, Harrak MJ, de Hoog GS (2007). Spectrum of Clinically relevant *Exophiala* species in the United States. J Clin Microbiol.

[7] Özhak-Baysan B, Öğünç D, Döğen A, Ilkit M, de Hoog GS (2015). MALDI-TOF MS-based identification of black yeasts of the genus *Exophiala*. Med Mycol.

[8] Fraser M, Brown Z, Houldsworth M, Borman AM, Johnson EM (2015). Rapid identification of 6328 isolates of pathogenic yeasts using MALDI-ToF MS and a simplified, rapid extraction procedure that is compatible with the Bruker Biotyper platform and database. Med Mycol.

[9] Pinto A, Halliday C, Zahra M, van Hal S, Olma T, Maszewska K (2011). Matrix-assisted laser desorption ionization-time of flight mass spectrometry identification of yeasts is contingent on robust Rreference spectra. PLoS ONE.

[10] Nagano Y, Elborn JS, Millar BC, Goldsmith CE, Rendall J, Moore JE (2008). Development of a novel PCR assay for the identification of the black yeast, *Exophiala* (*Wangiella*) *dermatitidis* from adult patients with cystic fibrosis (CF). J Cyst Fibros.

[11] Ergin Ç, Gök Y, Bayğu Y, Gümral R, Özhak-Baysan B, Döğen A (2016). ATR-FTIR spectroscopy highlights the problem of distinguishing between *Exophiala dermatitidis* and *E. phaeomuriformis* using MALDI-TOF MS. Microb Ecol.

[12] Steinmann J, Schmidt D, Buer J, Rath PM (2011). Discrimination of *Scedosporium prolificans* against *Pseudallescheria boydii* and *Scedosporium apiospermum* by semiautomated repetitive sequence-based PCR. Med Mycol.

[13] Rath PM, Müller KD, Dermoumi H, Ansorg R (1997). A comparison of methods of phenotypic and genotypic fingerprinting of *Exophiala dermatitidis* isolated from sputum samples of patients with cystic fibrosis. J Med Microbiol.

[14] Packeu A, Lebecque P, Rodriguez-Villalobos H, Boeras A, Hendrickx M, Bouchara J-P, Symoens F (2012). Molecular typing and antifungal susceptibility of *Exophiala* isolates from patients with cystic fibrosis. J Med Microbiol.

[15] Centers for Disease C, Prevention. *Exophiala* infection from contaminated injectable steroids prepared by a compounding pharmacy-United States, July-November 2002. MMWR Morb Mortal wkl Rep. 2002;51(49):1109–12. 12530707

[16] Vasquez A, Zavasky D, Chow NA, Gade L, Zlatanic E, Elkind S (2017). Management of an outbreak of *Exophiala dermatitidis* bloodstream infections at an outpatient oncology clinic. Clin Infect Dis.

